# Novel Fluorescein Angiography-Based Computer-Aided Algorithm for Assessment of Retinal Vessel Permeability

**DOI:** 10.1371/journal.pone.0061599

**Published:** 2013-04-23

**Authors:** Yonatan Serlin, Geva Tal, Yoash Chassidim, Yisrael Parmet, Oren Tomkins, Boris Knyazer, Alon Friedman, Jaime Levy

**Affiliations:** 1 Department of Physiology and Neurobiology, Zlotowski Center for Neuroscience, Ben-Gurion University of the Negev, Beer-Sheva, Israel; 2 Department of Biomedical Engineering, Zlotowski Center for Neuroscience, Ben-Gurion University of the Negev, Beer-Sheva, Israel; 3 Department of Industrial Engineering and Management, Zlotowski Center for Neuroscience, Ben-Gurion University of the Negev, Beer-Sheva, Israel; 4 Moorfields Eye Hospital, London, United Kingdom; 5 Royal Surrey County Hospital, Guildford, United Kingdom; 6 Department of Ophthalmology, Soroka University Medical Center and Faculty of Health Sciences, Ben-Gurion University of the Negev, Beer-Sheva, Israel; 7 Institute of Neurophysiology, Neurocure Research Center, Charité Universitätsmedizin, Berlin, Germany; University of Tennessee, United States of America

## Abstract

**Purpose:**

To present a novel method for quantitative assessment of retinal vessel permeability using a fluorescein angiography-based computer algorithm.

**Methods:**

Twenty-one subjects (13 with diabetic retinopathy, 8 healthy volunteers) underwent fluorescein angiography (FA). Image pre-processing included removal of non-retinal and noisy images and registration to achieve spatial and temporal pixel-based analysis. Permeability was assessed for each pixel by computing intensity kinetics normalized to arterial values. A linear curve was fitted and the slope value was assigned, color-coded and displayed. The initial FA studies and the computed permeability maps were interpreted in a masked and randomized manner by three experienced ophthalmologists for statistical validation of diagnosis accuracy and efficacy.

**Results:**

Permeability maps were successfully generated for all subjects. For healthy volunteers permeability values showed a normal distribution with a comparable range between subjects. Based on the mean cumulative histogram for the healthy population a threshold (99.5%) for pathological permeability was determined. Clear differences were found between patients and healthy subjects in the number and spatial distribution of pixels with pathological vascular leakage. The computed maps improved the discrimination between patients and healthy subjects, achieved sensitivity and specificity of 0.974 and 0.833 respectively, and significantly improved the consensus among raters for the localization of pathological regions.

**Conclusion:**

The new algorithm allows quantification of retinal vessel permeability and provides objective, more sensitive and accurate evaluation than the present subjective clinical diagnosis. Future studies with a larger patients’ cohort and different retinal pathologies are awaited to further validate this new approach and its role in diagnosis and treatment follow-up. Successful evaluation of vasculature permeability may be used for the early diagnosis of brain microvascular pathology and potentially predict associated neurological sequelae. Finally, the algorithm could be implemented for intraoperative evaluation of micovascular integrity in other organs or during animal experiments.

## Introduction

Fluorescein angiography (FA) is widely used as the basis for diagnosis, treatment consideration and follow-up of retinal pathologies, especially diabetic retinopathy and age-related macular degeneration, two leading causes of blindness [1]. Abnormal fluorescence patterns represent pathological blood flow or altered vessel permeability. Leakage patterns represent disruption of the inner blood-retinal barrier (BRB) as well as neovascularization. A growing body of evidence emphasizes that pathological vessels are not limited to the retina and that similar alterations occur in small vessels within the brain. Recent studies in experimental models, as well as clinical trials, stress the key role of microvessel pathology and specifically blood-brain barrier (BBB) dysfunction in different neurological disorders, including neurodegenerative and neuro-immune diseases, stroke and its complications, epilepsy as well as other cognitive and mood disorders [2]–[6]. Despite the growing interest in retinal microvessels as early predictors for ocular and brain diseases, to date, the use of FA for the detection of microvessel alterations is limited due to a lack of objective and acceptable quantitative analysis methods for vessel structure and function. This in turn, raises issues regarding standardization of subsequent pathology evaluation or by different experts. Establishing a quantitative, objective and reliable image analysis tool, based on readily available FA studies, may prove to be a non-invasive method for the diagnosis of retinal diseases, as well as a potential predictor for central nervous system pathologies.

We have recently established a method for quantitative assessment of brain vessel permeability using FA in experimental rodent models [7]. Based on the results obtained from such models, we were able to evaluate the accuracy of this new analysis method and apply it in a clinical setting. The objectives of the present study were to (1) implement similar algorithms to assess vessel permeability in retinal FA examinations as part of a new platform for computer-aided diagnosis of retinal images (RetiCAD); (2) test the method in cohorts of healthy individuals and patients diagnosed with diabetic retinopathy; and (3) perform a preliminary evaluation for clinical significance by comparing the results obtained using RetiCAD to those of the standard examination (the “gold standard”).

## Materials and Methods

### Subjects and Study Design

Twenty-one subjects took part in this study, 13 patients (mean age±SD was 55.9±8.19 years, range 41–69) with diagnosed diabetic retinopathy and 8 healthy volunteers (mean age 29.5±3.7 years, range 25–36,). Patients were randomly selected from those attending the retina clinic without any information regarding their degree of retinopathy (i.e. equal probability of selection method (EPSEM)). Retrospective examination of the patients clinical records revealed bilateral moderate non-proliferative diabetic retinopathy (NPDR) (n = 2 patients), severe NPDR (n = 8) and proliferative diabetic retinopathy (PDR) (n = 3). In order to create a permeability range for normal retinal vasculature and a pathological threshold, and to exclude potential age-related changes, healthy young adults were chosen as controls. The protocol for this study was approved by the Soroka University Medical Center Ethics Committee; a written informed consent was obtained from all participants. For each subject FA retinal images were acquired according to a modified clinical protocol: images were captured from time ‘0’ (intravenous injection of sodium fluorescein (5 ml, 20%)) using a Topcon 50EX camera (excitation wavelength between 465–490 nm, emission of 520–530 nm; OIS WinStation 5000™ software (Ophthalmic Imaging Systems, Sacramento, CA)). During the first 90 seconds, images were taken from one, pre-selected eye with prominent retinal pathology (as indicated by the treating ophthalmologist) at a rate of 20 images/min. During the next 8′30′′, images were obtained at the highest possible rate from both eyes (average of 5 images/min from each eye). The same protocol was applied for one random eye for each healthy subject. In total, 21 eyes were included in the analysis.

### Description of the Analysis Algorithms

Based on our established method for quantitative assessment of blood-brain barrier permeability using FA in animal experiments [7], the analysis of the FA data was done using in-house MATLAB based software. Data pre-processing included automatic classification to right/left eye images by detection of the optic disc center-of-mass and exclusion of noisy and none-retinal images (e.g., due to movement). To achieve anatomical alignment of consecutive images, the FA series of the selected eye were co-registered using 2D sub-pixel registration [8]. In short, registration was performed using the phase correlation method which calculates the cross correlation between two images. Since registration is always performed between a set of two images, the registration of the entire sequence was achieved by sequential registration of consecutive images, starting from a reference image with the maximal correlation to the average of the complete series of images. The first two images to be registered were the one before and the one following the reference image; the second couple was registered to the previously chosen and so forth. Edge detection filter was applied to each image before registration using a LOG (Laplacian of Gaussian) 2D filter of size 8×8 pixels with standard deviation σ = 1.4. These pre-processing steps allowed pixel based analysis of the data in both, spatial (anatomy) and temporal (dynamics) dimensions. Each pixel could then be characterized by its temporal behavior by extracting and analyzing the intensity values over time.

### Measuring Permeability

**Figure 1:**
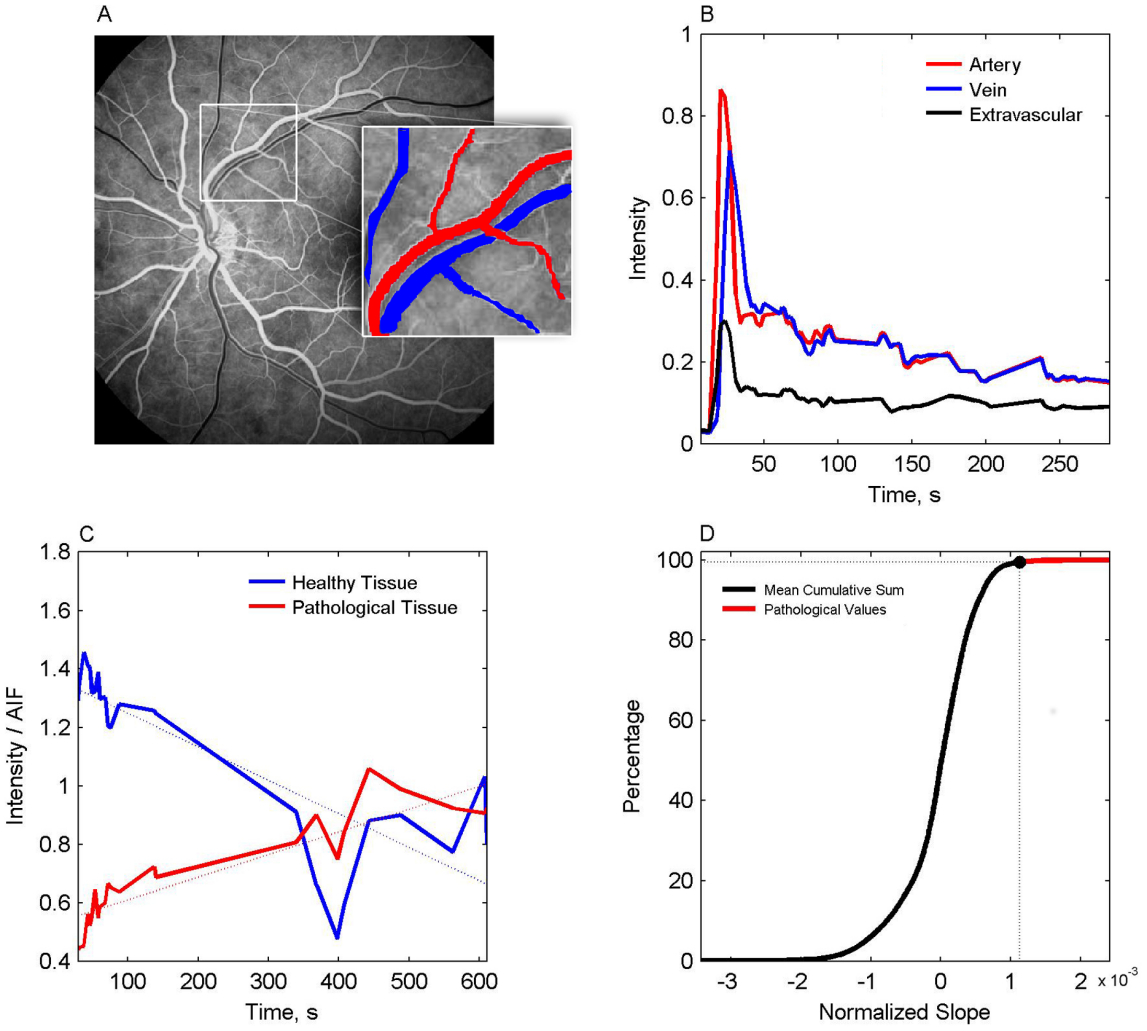
Analysis of FA images. Using a registered averaged image (A), a region of interest within a retinal artery is selected (red in inset) to compute an arterial input function. The plots in (B) demonstrate changes in fluorescent intensity within consecutive images in an artery (red), vein (blue) and extravascular region (black). (C) A linear fit (dashed line) was fitted to the late phase of the FA study, showing the normal decline (negative slope) obtained in a healthy tissue, and a positive slope (dye accumulation) in a pathological region with permeable vessels. (D) Averaged cumulative histogram for all healthy controls. Threshold for pathological values was chosen as the slope above 0.05% of the pixels in healthy individuals.

**Figure 2:**
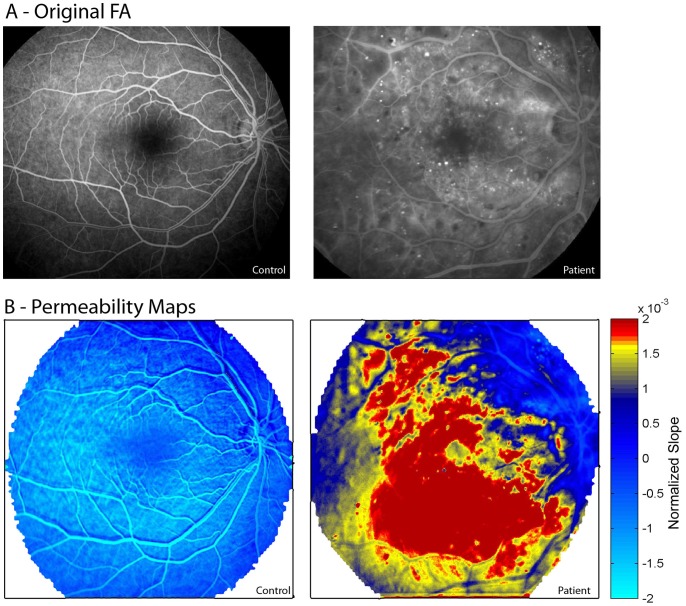
Generation of permeability maps. (A) Original registered averaged FA image from a healthy individual (left) and a patient diagnosed with proliferative diabetic retinopathy (right). (B) Computed permeability maps for the same individuals as in (A). Note the higher permeability values in the patient’s retina.

Quantification of vessel permeability was performed by observing the temporal changes in intensity for each pixel in the registered images sequence which were normalized and plotted (Fig. 1B). Normalization was achieved by dividing the intensity of each pixel at a given image (i.e. time point) with the mean intensity of the major arteries exiting the optic disk (manually selected), representing the arterial input function (AIF). A linear curve was fitted for the change in normalized intensity over time, from which the slope was extracted (Fig. 1C). The rationale behind this measure is based on the assumption that after fluorescein injection healthy blood vessels and retinal tissue demonstrate a rise in intensity followed by a steady decline due to fluorescein wash out. Vascular permeability changes will result in accumulation of the dye, with an increase in relative intensity over time and a positive slope. To establish a threshold for distinguishing pathological from healthy tissue, the values from all healthy controls were plotted in a cumulative sum plot. The value measured at the top 0.5% was chosen as the maximal value for a “normal” slope of healthy retina (Fig. 1D). In the RetiCAD computed permeability maps, pixels with slope values below the 99.5% determined threshold (“normal slope”) were assigned as blue, while pixels exceeding this value (“pathological slope”) were assigned as yellow to red (Fig. 2). The details of the RetiCAD algorithm and its implementation are described in previous publication [7] and above. RetiCAD MATLAB scripts are freely available for download at http://fohs.bgu.ac.il/neurophysio/project.rar.

### Diagnosis and Evaluation of FA Images and Permeability Maps

Three experienced ophthalmologists independently evaluated the 21 FA series for the presence of permeability changes, by indicating and localizing permeable areas on a 3×3 orthogonal grid (i.e. 9 squares). The FA images were randomized and masked as to the identity and health status of the subjects. Each specialist rated the permeability state of the vessels in each of the 9 squares and an overall diagnosis for each subject. Subsequently, on a separate occasion, also in a masked, randomized manner, each rater received 21 permeability maps computed as described above, from the same FA images, masked for the identity of the patient. Evaluation of the RetiCAD maps was carried out in the same fashion as that for the original FA series.

### Statistical Analysis and Validation

Statistical analyses were performed with SPSS 17.0 (SPSS Inc., Chicago, IL) and R statistical software (R Development Core Team, 2007). Fleiss’ kappa coefficient was calculated for rater’s agreement on the diagnosis of the initial FA image and the evaluation of the computed maps, to measure inter-rater agreement beyond chance. Comparison of correlated kappa statistics was done using the bootstrap technique [9]. The results of the two image analysis methods were dichotomized for the primary comparison and the diagnosis was made between pathologic or non-pathologic subject. Kappa statistics were interpreted based on the convection statistical analysis ranges [10]. Sensitivity and specificity were calculated for the permeability maps capability to affirm the distinction between an individual known to suffer from increased vessel permeability and a healthy subject. Angiography is considered the “gold standard” for evaluation of the retinal vasculature. For obtaining the most accurate FA diagnoses we used the diagnostic results of the rater that had the highest agreement with the known medical diagnosis according to medical records (simple kappa coefficient = 1.0). Assessment of the method to reveal and localize areas of increased vascular permeability over a 3×3 orthogonal grid was done using a one-tailed McNemar’s test. The level of statistical significance was set at *P*<0.05. Each square of the grids received a score, according to the overall consensus for that square. Perfect agreement received the score of ‘1’ when all 3 raters agreed that in a specific square there are no permeability changes or all 3 raters agreed that permeability changes do exist. Any inconsistency in rater’s decision for a specific square received the score of ‘0’. To deduce the total consensus of the 3 raters per eye, we used the median value of the overall 9 squares score.

## Results

**Figure 3:**
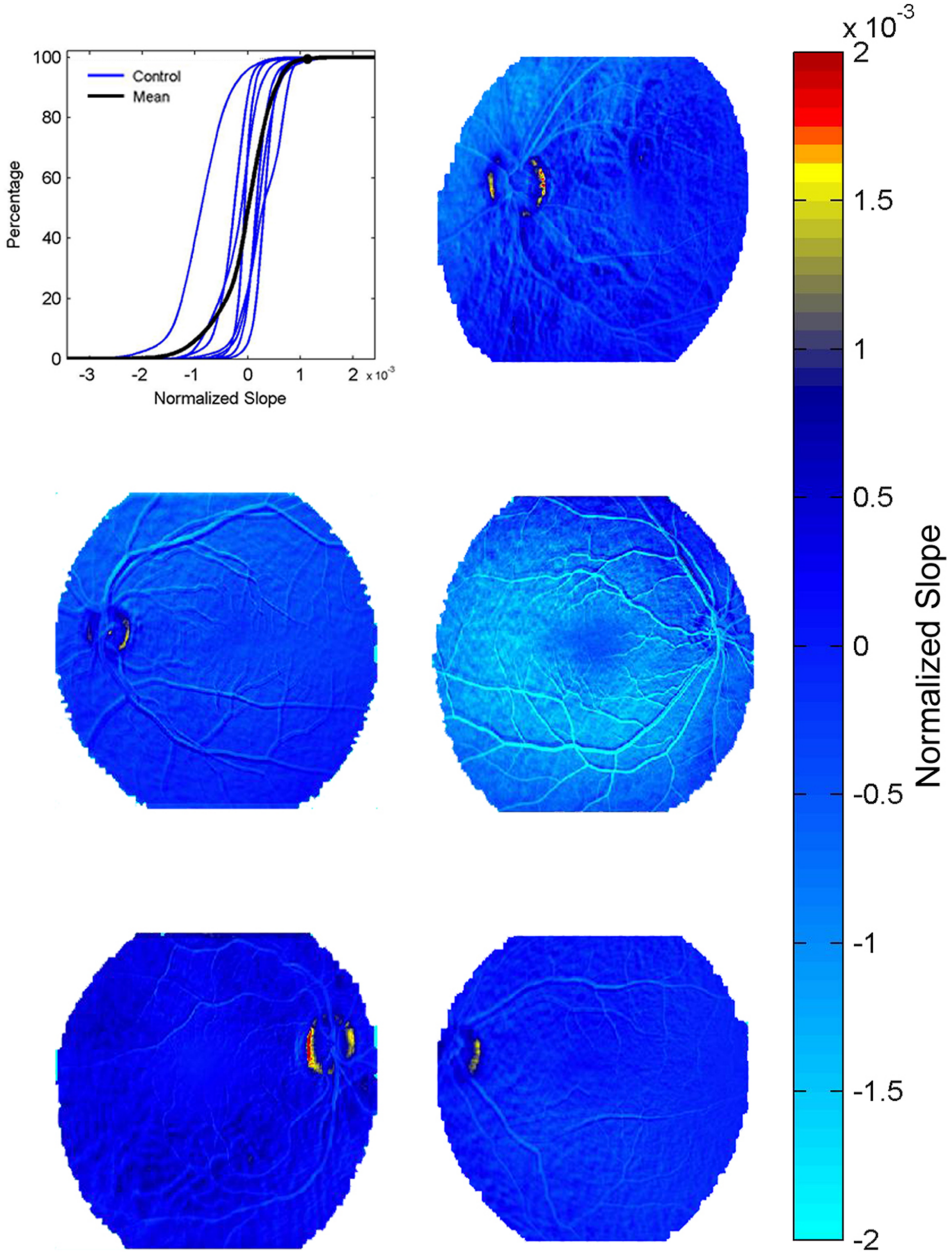
Permeability maps in healthy subjects. Cumulative histogram of permeability values showing the averaged curve (black curve) of eight healthy volunteers (blue lines). Note the similar range of values for all controls. Retinal maps of 5 healthy controls are presented. Note that in all control, “high” values (yellow-red) are rare and are concentrated around the optic nerve.

**Figure 4:**
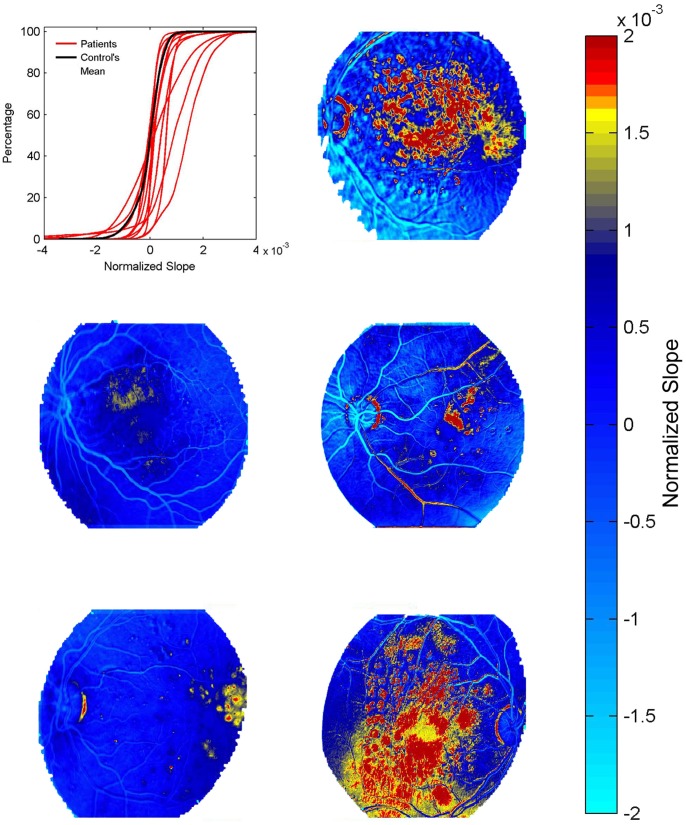
Permeability maps for the patients’ group. Cumulative histogram of permeability values showing the averaged curve for the healthy group (black curve) and 13 curves for the individual patients (red lines). Note the “right shift” of the cumulated graph in most patients, associated with large number of pixels showing abnormally high values (yellow-red) in different regions of the retina. Retinal maps of 5 patients are presented.

Eight healthy controls underwent FA imaging and permeability maps were computed for each. The cumulative histogram for the slope values was similar among the healthy controls (Fig. 1D, Fig. 3), suggesting that inter-individual physiological differences, small differences in the dose of the injected dye or image acquisition procedures did not affect slope values. This allowed us to set an arbitrary maximal slope value (above 99.5% of the values derived from the averaged cumulative histogram, Fig. 1D) characterizing permeability within the “healthy retina”. In healthy volunteers, slope values above the defined maximum (>0.0011) were observed in 7 out of the 8 eyes (87.5%) and were spatially limited to the optic disc (Figs. 2–3). Conversely, computed maps of all thirteen patients diagnosed with retinal pathology clearly showed higher percentage of pixels with abnormal values (15.5 vs. 0.5% in patients and controls, respectively), which were found in all regions of the retina (Fig. 4). In healthy volunteers, the range of the normalized slope values (excluding the optic disc region that was manually segmented) was between −0.0011 to +0.00069, while in patients pixels’ values ranged between −0.001 to +0.0026. Thus, while the lower slope value was similar, the upper permeability value was 3.8-fold greater in patients compared to healthy controls.

Based on the kappa statistical analysis range, agreement among the raters was “substantial” for the evaluation of the original FA studies (Fleiss’ kappa 0.795 (*P<0.001*)), and “almost perfect” for the evaluation of the RetiCAD maps (Fleiss’ kappa 0.857 (*P<0.001*)). The notable improved agreement was found to be not statistically significant (95% confidence interval for the difference in kappa (−0.394 to 0.265)). The average sensitivity and specificity of the computed maps were 0.974 and 0.833 respectively, representing its discrimination capabilities. The efficacy of the permeability maps in revealing and localizing areas of increased vascular permeability was tested against the rater’s conclusion obtained during the interpretation of the original FA images. A one-tailed McNemar’s test showed that the use of the permeability maps appears to give a statistically significant improved consensus among raters for their overall conclusion for each subject (*N = 21, P = 0.013*).

## Discussion

Fundus fluorescein angiography has been used for more than 50 years [11]. Recent marked improvements in informatics together with advances in imaging and digital photography have revolutionized the field of retinal imaging. However, methods for computerized analysis of retinal angiograms are not yet established for routine use in the diagnosis of retinal pathologies, though marked improvements were accomplished (e.g., [12]–[14]).

FA is still a subject of great variability even among experienced readers [15]. Our newly developed approach showed high sensitivity and specificity rates and enabled detection and quantification of regions with increased retinal permeability. Consensus among raters was demonstrated to significantly increase when diagnosis was based on the new permeability maps, rather than conventional FA diagnosis.

This novel approach was based on a previously established FA-based method for the quantitative assessment of brain vessel permeability performed in animal studies [7]. The use of quantitative approaches in experimental conditions that induce endothelial damage confirmed their potential to detect small, visually undetected changes in blood flow and permeability. The similarities between brain and retinal vessels and their respective barriers, made it possible to adjust the methods to the retinal FA data in terms of signal values, as well as spatial and temporal resolution. Pre-processing was required to exclude technically poor images and re-align images to allow the computation of dynamic changes of fluorescent intensity within the same pixel. Furthermore, using an arterial input function as a normalization reference allowed early detection of regions with subtle leakage. The protocol used in this study was similar to the standard clinical FA protocol routinely applied in many eye clinics, suggesting the clinical applicability of the method. An important issue is the high sampling rate required within the first few seconds after injection, which improve the normalization to the arterial input function. Thus, while future studies are awaited to define the optimal acquisition protocol, it seems likely that using video capabilities will give even more accurate and reliable permeability values.

One clear limitation of the present study is the small number of cases. Yet, it revealed and localized the areas of increased vascular permeability adequately and consistently compared with the manual grading by three masked raters. While a relatively small range of permeability values was found within our healthy controls, we did not examine the possible differences related to gender, age and non-retinal diseases commonly occurring in the aged population (e.g., hypertension). Future, larger clinical studies are needed to confirm our results in a large number of patients with different vascular pathologies. In addition, the presented algorithm may demonstrate a significant advantage in early detection and follow-up of retinal disease progression and response to treatment. A further outcome of this method may provide quantitative information of vessel anatomy, including measures for caliber, density and tortuosity.

In summary, this newly developed algorithm has the potential of being faster, objective, sensitive and more accurate than the present subjective physician diagnosis of patients with retinal pathology. Future perspectives include the assessment of new treatments as well as the prediction and prevention of neurological complications in patients with small vessel diseases.
